# Plant-Based Nano-Delivery Systems in the Treatment of Inflammatory Disorders

**DOI:** 10.3390/pharmaceutics18020150

**Published:** 2026-01-23

**Authors:** Catarina R. Silva, Amélia C. F. Vieira, Ana Cláudia Paiva-Santos, Francisco Veiga, Gustavo Costa

**Affiliations:** 1 Department of Pharmaceutical Technology, Faculty of Pharmacy of the University of Coimbra, University of Coimbra, 3000-548 Coimbra, Portugal; cata.rajado@gmail.com (C.R.S.); ameliavieira@ff.uc.pt (A.C.F.V.); acsantos@ff.uc.pt (A.C.P.-S.); fveiga@ci.uc.pt (F.V.); 2 LAQV/REQUIMTE, Department of Pharmaceutical Technology, Faculty of Pharmacy of the University of Coimbra, University of Coimbra, 3000-548 Coimbra, Portugal; 3 Coimbra Institute for Clinical and Biomedical Research (iCBR), Faculty of Medicine, CNC.IBILI Consortium and CIBB Consortium, University of Coimbra, 3000-548 Coimbra, Portugal

**Keywords:** inflammation, anti-inflammatory, medicinal plant, natural compound, nanotechnology, nanosystem, nanocarrier

## Abstract

Inflammation is strongly related to the development of multiple chronic diseases, such as cardiovascular and autoimmune diseases, and is considered a crucial target for new therapeutic approaches, since it significantly impacts public health, contributes to high mortality rates, and decreases the quality of life. Conventional anti-inflammatory approaches are commonly used, but they present multiple limitations, such as undesirable side effects and low target-specificity. Medicinal plants and their bioactive phytochemical compounds have been studied in recent years and are considered promising alternatives to classical therapies. They are widely recognized for their capacity to modulate inflammatory pathways, regulate inflammatory responses, and consequently reduce inflammation and related symptoms. Although they are considered a good therapeutic alternative, their application in the human body is limited by certain characteristics, such as low solubility, which leads to rapid metabolism and excretion by the organism, significantly reducing bioavailability; for these reasons, the use of medicinal plants remains a biopharmaceutical challenge. Nanotechnology represents a promising tool in this context, since it can improve several characteristics of these compounds. By incorporating plant-derived compounds in nanosystems, considerable advantages, including sustained release, protection from degradation, an increase in the specificity to target tissues, and consequent reduction in toxicity, can be achieved. Thus, nanosystems promote more favorable therapeutic outcomes. This work aims to compile scientific evidence supporting the use of medicinal plants and their bioactive phytochemical compounds, incorporated in nanosystems, in inflammatory disorders. This review enlarges knowledge by integrating both in vitro and in vivo studies involving multiple medicinal plants and bioactive phytochemical compounds, describing their mechanisms of action and the nanosystems employed for drug delivery. In the future, the need for deeper mechanistic studies, the development of targeted and stimuli-responsive systems, and advancement toward clinically translatable, sustainable, and cost-effective plant-based nanotherapies is required.

## 1. Introduction

Inflammation is a strongly dynamic process that functions as a natural defense mechanism. It is the first protective response of the body’s immune system when triggered by irritants, microbial invasions, antigens, damaged cells, pathogens, or other harmful stimuli [1,2]. It is known that inflammation contributes significantly to the occurrence and progression of numerous chronic diseases, including diabetes, cancer, cardiovascular diseases, obesity, and eye disorders [1,3]. Furthermore, inflammation plays a central role in the pathophysiology of autoimmune diseases (for example, arthritis or inflammatory bowel disease). While inflammation is an essential physiological defense mechanism, if persistent or severe, it can lead to disease [4].

Inflammation can be classified as acute or chronic, depending on the cellular mechanisms involved [1]. Acute inflammation is a short process, lasting from minutes to a few days, and is characterized by the exudation of fluid and plasma proteins as well as the migration of leukocytes into extravascular compartments. On the other hand, chronic inflammation is an enduring response that can happen when the triggering factor cannot be eliminated or when tissue repair at the site of injury is not effective. This process is characterized by the persistence of an inflammatory reaction and simultaneous tissue destruction and repair [5].

Cytokines involved in inflammation processes can be categorized into two sub-groups: pro-inflammatory and anti-inflammatory. Pro-inflammatory cytokines promote and develop inflammation and immune responses. Some examples include tumor necrosis factor-alpha (TNF-α), interleukins such as interleukins IL-1, IL-6, and IL-12, as well as interferon-alpha (IFN-α) and interferon-gamma (IFN-γ). In contrast, anti-inflammatory cytokines, including interleukins Interleukin-4 (IL-4), Interleukin-10 (IL-10), Interleukin-13 (IL-13), and transforming growth factor-beta (TGF-β), are capable of suppressing inflammation and regulating immune tolerance [6]. To maintain a healthy immune system and prevent inflammatory diseases, it is crucial to keep the balance between pro-inflammatory and anti-inflammatory cytokines [7].

In extreme cases, inflammation can have severe clinical consequences and can be severely prejudicial to individuals; it is a leading factor in morbidity and mortality worldwide [8]. Moreover, inflammation also constitutes a major healthcare burden, contributing significantly to health costs; for instance, a study by Linschoten and colleagues shows that the healthcare costs of inflammatory bowel disease have been increasing, resulting in extremely high costs [9]. For these reasons, inflammation constitutes a key therapeutic target for the development of innovative strategies and pharmacological interventions [2].

Since ancient times, medicinal plants have been used to treat multiple diseases. Many of their bioactive compounds are able to modulate inflammatory pathways and, consequently, decrease inflammatory reactions. Their use not only increases positive therapeutic outcomes but also reduces complications and adverse effects [2]. Since they offer anti-inflammatory potential, safety, and reduced side effects, these compounds are being deeply investigated and are considered promising candidates for managing inflammation [8].

Despite their therapeutic potential, many natural compounds face limitations such as low solubility, stability, and bioavailability [10]. Also, the clinical outcomes of inflammatory disorders are not considered adequate, so it urges to develop innovative strategies to address inflammation, and that is achievable by merging traditional therapeutics with innovative technologies [3]. In this context, nanotechnology plays an important role. Following the definition of the National Nanotechnology Initiative, nanotechnology is the understanding and control of matter at the nanoscale, at dimensions between 1 and 100 nanometers, given that in these dimensions, material properties can differ significantly from those at larger scales, offering other opportunities for adjustment [11].

Nanotechnology, which is considered one of the most important scientific achievements of the century, has multiple applications in the health field, such as diagnostics, transport, and drug delivery [12], tissue engineering, and antimicrobials [13], and it has witnessed extraordinary advancements in recent years [14].

Producing nanosystems that carry medicinal plants has been a growing practice, and it is becoming extensively applied. It is considered that it can innovate and transform the therapeutic field, giving it a new perspective [14]. Nanotechnology offers targeted, more precise, and safe delivery, which increases the efficiency in carrying compounds. With this type of delivery, there is a higher proportion of the drug reaching the target site and less exposure of non-specific sites, reducing systemic exposure, improving both efficacy and safety, and decreasing side effects [15].

Plant-derived nanoparticles stand out as particularly promising drug candidates due to their unique and millenary anti-inflammatory properties [16]. These systems have shown particular promise in pharmaceutical technology, where they enhance the solubility and gastrointestinal stability of poorly soluble phytochemicals such as curcumin, resveratrol, and flavonoids [17]. However, plant-based nanosystems are often limited by batch-to-batch variability, reduced physicochemical stability, lower drug-loading capacity, and comparatively limited control over release kinetics and targeting efficiency [18]. Nonetheless, plant-based nano-delivery systems provide a safer and more sustainable platform for the delivery of natural bioactive compounds [19].

There are different types of nanosystems, such as nanoparticles, nanogels, extracellular vesicles, polycyclodextrins, nanoliposomes, nanosheets, hyalurosomes, electrospinning dressings, and self-nano-emulsifying systems.

Nanoparticles are particles with a nanometric size that are engineered at the atomic or molecular level and are usually found in a spherical morphology [20]. Nanoparticles demonstrate important properties at many levels, including structural, biological, chemical, and mechanical [21].

Nanogels are nanometric structures that possess properties of both hydrogels and nanoparticles. They operate by allowing water absorption while maintaining structural integrity and offer effective drug-loading capability and stability [22].

Extracellular vesicles are also considered a good option in drug delivery, due to their physical functions; they are nanosized exosomes, which have 70 to 150 nanometers [23].

Cyclodextrins are systems that can form a reversible inclusion complex with guest molecules. They possess many important properties, including biocompatibility, low immunogenicity, and safety; thus, they represent innovative nanocarriers with great potential [24,25].

Nanolipossomes represent an advanced technology for the encapsulation and delivery of bioactive agents. These systems have a bilayer structure, which is highly compatible with the skin surface, a characteristic that facilitates enhanced permeation to the intended site [26]. A variety of compounds can be incorporated into these systems, which can enhance their performance by improving their solubility, stability, and bioavailability, while also preventing unwanted interactions with other molecules [27].

Nanosheets are ultrathin films that, due to their high adhesion and flexibility, are considered promising options for use in biomedical fields. They possess a distinctive electronic structure with atomic thickness forming two-dimensional sheets that exhibit a high surface-area-to-thickness ratio [28,29,30].

Hyalurosomes are composed of hyaluronic acid, a highly efficient biopolymer for biomedical applications, due to its biodegradability and outstanding biological properties. In the nanofiber form, this polymer can mimic biological membranes, including skin layers [31]. Electrospinning is a nanofiber fabrication technique, recognized for its simplicity, cost-effectiveness, and versatility, that has been widely used. This flexible process offers unique advantages for producing uniform nanofibers and fabrics from nearly any synthetic polymer, as well as many natural polymers, with adjustable pore structures [31,32].

Plant-based nano-delivery systems function through a common mechanism that involves the encapsulation, protection, transport, controlled release, and biological uptake of bioactive compounds. These nanocarriers encapsulate bioactive molecules via hydrogen bonding, hydrophobic interactions, or electrostatic forces, thereby improving aqueous dispersibility and physicochemical stability of poorly soluble compounds [33]. Due to their nanoscale size and surface characteristics, these systems enhance membrane adhesion and promote interaction with biological barriers, facilitating improved absorption through endocytosis or paracellular transport pathways [34]. Furthermore, these systems can be engineered to respond to physiological stimuli such as pH variation or enzymatic activity, enabling site-specific and sustained release of encapsulated bioactive compounds in targeted regions of the gastrointestinal tract or at cellular targets [5]. Following cellular uptake and intracellular release, the nanocarriers undergo enzymatic degradation into non-toxic metabolites, ensuring safe clearance from the body [35]. These new nanotechnologies applied to natural compounds or extracts are promising, but they lack standardization, source variability, scalability, mechanistic clarity, and robust safety data [36,37,38]. Also, a regulatory framework is an imperative that needs to be addressed before an effective and safe clinical translation [3].

Self-nano-emulsifying drug delivery systems are a promising strategy created to overcome the issues associated with insufficient water solubility and oral delivery of drug molecules. They are composed of an oil phase, surfactant, and cosurfactant or cosolvent [39].

However, nanoparticle exposure may induce harmful interactions and other subsequent mechanisms that can potentially result in adverse effects and nanotoxicity. One of the main findings is that size, shape, surface area, and chemical composition of NPs are key determinants of their toxicity [40]. Also, dosage regimen, route of exposure, surface chemistry, degree of aggregation, transmembrane diffusion, and excretion pathway play key roles in contributing to nanotoxicity [41].

In its current state, nanoparticle toxicity evaluation requires careful case-by-case assessment, but it can be categorized into neuro-, respiratory, cardiovascular, endocrine, reproductive, geno-, carcino-, and immunotoxicity [42]. Genotoxicity, which is responsible for damage to the genetic material, is crucial since it leads to deep disturbances, including cancer. Recent studies have undoubtedly brought substantial progress in the approaches to investigating the genotoxicity of NPs. Although no firm guidelines exist regarding the most suitable tests needed to acceptably establish safety from the potential genotoxicities of NPs, it has become clearer that gene or chromosomal mutations should be considered [43]. In vitro studies point out that nanomaterials can cause DNA damage by direct interactions, ROS generation, and cellular disruption [44]. Studies on the potential immunotoxic effects of nanoparticles remain very limited. Most nanosystems can only target the inflammatory sites rather than specific types of immune cells, leaving room for many undesired effects [45].

The clinical implementation of plant-based nano-delivery systems to treat inflammatory disorders remains at an early translational stage, despite substantial preclinical evidence supporting their therapeutic potential. Clinical evidence is largely limited to a small number of human studies involving phytosome-based or nano-encapsulated plant bioactive compounds, which have reported to improve symptoms management and to reduce inflammatory biomarkers in conditions such as osteoarthritis and rheumatoid arthritis, showing favorable safety and adequate tolerability profiles [46]. Notably, most of these formulations are commercialized as nutraceuticals or dietary supplements rather than approved nanomedicines, reflecting ongoing regulatory and translational challenges. These challenges include variability in plant raw materials, lack of standardized large-scale manufacturing processes, limited pharmacokinetic and long-term safety data, and the absence of clear regulatory frameworks specific to plant-derived nanocarriers. Consequently, while plant-based nano-delivery systems represent a promising and safe strategy for inflammation management, particularly for chronic diseases requiring long-term therapy, their clinical use is currently confined to adjunct or supportive roles, with further well-designed randomized clinical trials and regulatory harmonization required for broader pharmaceutical adoption [47].

This work aims to integrate relevant information of medicinal plants and bioactive phytochemical compounds application in the treatment of inflammatory conditions, focusing on nanotechnological strategies. Throughout this work, the collected data on extracts and natural compounds obtained from medicinal plants, studied in both in vitro and in vivo models, are presented with their mechanisms of action and anti-inflammatory effects in different diseases.

## 2. Method

### 2.1. Source and Search Strategy

A literature search was conducted using the online database PubMed. The search strategy involved the use of various combinations of keywords such as “medicinal plant”, “nanotechnology”, “inflammation”, “anti-inflammatory”, “nanosystem”, “nano”, “nano-carrier”, “nanomedicine”, “nano-based”, “nano-formulation”, “natural compound”, “natural product”, “herbal medicine”, and “anti-inflammation”. The articles were analyzed in terms of importance, and the most relevant ones were selected and included in this review.

### 2.2. Inclusion and Exclusion Criteria

Inclusion and exclusion criteria were established before the research to make it more precise and accurate. To be eligible for inclusion, the articles had to address the use of medicinal plants or their compounds, investigate anti-inflammatory effects, and incorporate a nanosystem for compound delivery. This monograph explores the anti-inflammatory effects of medicinal plants in multiple diseases with a nanosystem integrated; therefore, all the relevant articles that included important and useful information about those topics were included. Every article that did not mention these topics was excluded from the thesis.

The articles that included studies of natural compounds not derived from medicinal plants, articles that studied medicinal plants but did not approach inflammation conditions, and articles that did not incorporate a nanocarrier system were excluded. Additionally, only relevant scientific or review papers published between 2010 and 2025, and the studies related to these papers, were included.

## 3. Results and Discussion

The following sections summarize in vitro and in vivo studies conducted for various anti-inflammatory diseases and conditions, describing the investigated drugs and compounds and their mechanisms of action to attenuate inflammation. The following studies provide evidence that the selected medicinal plants and bioactive phytochemical compounds are capable of attenuating inflammation when delivered through nanotechnology-based formulations. Table A1 (Appendix A) provide detailed information related to the studies.

### 3.1. Acute Lung Injury

In a study addressing Acute Lung Injury (ALI), capsaicin was investigated in a nanosystem: capsaicin-iron nanoparticles. This nanosystem was studied in Lipopolysaccharide (LPS)-induced RAW 264.7 macrophage cells. They were divided into four groups: Control, LPS, Capsaicin + LPS, and Iron-capsaicin + LPS groups. This nanosystem demonstrated that it was able to decrease the levels of TNF-α and inducible Nitric Oxide Synthase (iNOS). In addition, the levels of TGF-β, through the Nuclear Factor kappa-light-chain-enhancer of activated B cells (NF-κB) pathway, increased and ameliorated inflammation [48].

In vivo experiments were performed using C57 mice, which were also divided into four groups: Control, LPS, Capsaicin + LPS, and Iron-capsaicin + LPS groups. After intraperitoneal injection, capsaicin-iron nanoparticles decreased the expression of pro-inflammatory mediators such as TNF-α and interleukin-6 (IL-6), controlling inflammation [48].

Another study concerning ALI consisted of the intraperitoneal administration of *Aloe vera*-loaded chitosan nanoparticles in rats (entrapment efficiency of 95.5 ± 1.25%). In the control group, the rats were administered a saline intraperitoneal injection before being infected with bacteria suspended in phosphate-buffered saline (PBS); in the *Staphylococcus aureus* group, the rats had an intraperitoneal injection of saline solution before being infected with *S. aureus*. In the *Aloe vera*-loaded chitosan nanoparticles group, rats were injected intraperitoneally with the nanoparticles before *S. aureus* infection. The nanoparticles could inhibit pro-inflammatory cytokines, such as Interleukin-1 beta (IL-1β), TNF-α, IFN-γ, IL-6, and NF-κB. There was also an increase in the level of mRNA of the cytokine IL-10, which has anti-inflammatory activity [49].

Curcumin and resveratrol nanoparticles (Cur-RV, loading capacity of ~70% and ~75%, respectively) were administered intranasally in a mouse model of ALI induced by LPS, and evidence confirmed their ability to decrease lung vascular permeability and reduce the levels of pro-inflammatory cytokines, such as TNF-α, IL-6, Intercellular Adhesion Molecule 1 (ICAM1), and iNOS. Cur-RV could modulate macrophage M1/M2 polarization; so, it resulted in a suppression of M1 polarization, which is pro-inflammatory, and promotion of M2 polarization, which is anti-inflammatory, consequently ameliorating inflammation [50].

In an in vitro study regarding ALI induced by LPS, naringin-loaded biomimetic nanoparticles were studied in RAW 264.7 cells and could reduce ROS production [51].

In an in vivo study, the same nanosystem was tested in mice with ALI induced by LPS. The mice were divided into 4 groups where they were intratracheally administered with PBS, naringin, naringin nanoparticles, and Cell Membranes–naringin nanoparticles. Especially in the last group, there was a reduction in IL-6 and IL-1β, relief of pulmonary edema, and polarization of macrophages towards M2 [51].

### 3.2. Benign Prostatic Hyperplasia

In an in vivo study, auraptene-loaded nanostructured lipid carrier (encapsulation efficiency of 67.5 ± 2.1%) was administered orally in rats, and it was compared to raw auraptene. The nanosystem could reduce the expression of inflammatory markers such as NF-kB, Interleukin 1-Beta (IL-1β), IL-6, and TGF-β. In addition, it could also enhance antioxidant activity [52].

### 3.3. Inflammation and Cancer

In the context of inflammation and cancer, curcumin–cholesteryl–hyaluronic acid nanogel (curcumin loading of ~7%) was tested in human pancreatic adenocarcinoma MiaPaCa-2 cells and could induce apoptosis and cytotoxicity in cancer cells with the suppression of NF-κB, TNF-α, and COX-2 [53].

This nanogel was also administered intravenously, intraperitoneally, and orally in two animal models: an orthotopic murine mammary 4T1 tumor model and a human pancreatic adenocarcinoma MiaPaCa-2 xenograft model. Mice were divided into three treatment groups: Control, Curcumin/dimethyl sulfoxide, and CHA CUR/saline. In those studies, there was an evident tumor growth inhibition using the nanogel [53].

Furthermore, *Anacardium occidentale*-loaded chitosan/polyvinyl alcohol (PVA)/Cu oxide nanoparticles were tested in COX-1 and COX-2 inhibition kits, while Ibuprofen was used as a positive control. A targeting and inhibition of both COX-1 and COX-2 activity was disclosed [15].

*Teucrium polium*-loaded solid lipid nanoparticles were incorporated into Ntra-2 cancer cells, compared to human foreskin fibroblasts, and there was a decrease in the expression of pro-inflammatory cytokines, such as IL-6 and IL-1β [54].

### 3.4. Liver Inflammation

In the context of drug-induced liver inflammation, celastrol, which was extracted from the roots of *Tripterygium wilfordii*, was loaded in Extracellular Membrane Vesicles (EMVs, loading capacity of 58.67 ± 0.79 μg), which were investigated in RAW 264.7 cells. There were four groups: Control, LPS, Celastrol, and Celastrol-EMV. The findings demonstrated that the nanosystem could modulate macrophage M1/M2 polarization. C-EMV can effectively downregulate TNF-α, IL-1β, and iNOS gene transcription, while upregulating Arg-1, Ym-1, and IL-10 gene transcription [55]. This nanosystem was also tested in mice through intravenous injection. There were five groups: Control, PBS, EMV, Celastrol, and Celastrol-EMV. C-EMV could modulate macrophage M1/M2 polarization, ameliorating inflammation. As a result, it suppressed the inflammatory response by reducing the pro-inflammatory cytokine levels, like TNF-α, IL-1β, iNOS, and preventing neutrophil infiltration [55].

Naringenin-reduced graphene oxide nanosheets (encapsulation efficiency of 68%) were administered orally in rats and could significantly decrease serum levels of pro-inflammatory cytokines, such as IL-1β and IL-6. The system ameliorated hepatic steatosis, insulin resistance, and oxidative stress, compared to free naringenin [56].

Ginsenoside compound K (CK)-loaded liver-targeted nanoparticles with albumin were tested in vitro in HepG2 cells, and there was a reduction in the expression of pro-inflammatory genes: cytokines like IL-16, IL-34, and chemokines such as CXCL5, CCL26, and CCL16 [57].

This nanosystem was also tested in vivo in high-fat diet-induced Non-Alcoholic Fatty Liver Disease (NAFLD) mice, through intravenous injection. There were four groups: Normal diet, Control, statin, and nabCK (the last three with a high-fat diet). With the nanoparticles, there was a retardation of the development of steatosis and fibrosis in mice and protection of cardiac tissues from lipotoxicity. In addition, there was an inhibition of the mTOR pathway [57].

### 3.5. Multiple Sclerosis

Regarding multiple sclerosis, engineered extracellular vesicles were loaded with Bryostatin-1 (entrapment efficiency of 56.83 ± 9.32%) and were tested in EAE mice through systemic injection. They ameliorated clinical disease development and inhibited inflammation by decreasing infiltration of pro-inflammatory cells, protecting the integrity of the blood–brain barrier, and altering the microglia pro-inflammatory phenotype [58].

### 3.6. Ocular Inflammation

β-carotene, a compound extracted from carrots, was encapsulated with hyaluronic acid-modified polycyclodextrins (drug loading of 8%) and was tested for its anti-inflammatory activity in Human Corneal Epithelial Cells (HCECs) in the context of ocular inflammation. The results demonstrated that the system reduced reactive oxygen species (ROS) levels and pro-inflammatory factors, including TNF-α, IL-1β, and leptin [59].

The nanosystem was also investigated in male SD rats through ocular instillation. There were six groups: Control, Model, Cyclosporine A, Beta-Carotene, PolyCycloDextrins@Beta-Carotene, and Hyaluronic Acid/PolyCycloDextrins@Beta-Carotene. In the last group, there was a reduction in inflammatory cytokines, such as TNF-α and IL-1β. There was an amelioration of the oxidative stress levels on the ocular surface and improvement of tear film stability. This treatment showed superior efficacy to Cyclosporine A, an FDA-approved first-line drug [59].

In an in vivo study, Licochalcone-A, isolated from the root of *Glycyrrhiza inflata*, was loaded into poly-(lactic-co-glycolic acid) (PLGA) nanoparticles (entrapment efficiency of 56.26 ± 0.16%) functionalized with PEG and cell-penetrating peptides (Tet-1 and B6): Lico-A PLGA-PEG-Tet1 (entrapment efficiency of 53.26 ± 0.62%) and Lico-A PLGA-PEG-B6 (entrapment efficiency of 31.36 ± 0.60%). The ocular inflammation model used to test the nanoparticles was New Zealand rabbits, and it was observed that those nanoparticles, through ocular administration, could reduce the ocular inflammation score, with PLGA-PEG-B6 showing superior results. In the study, the left eye was used as a control and received an isotonic saline serum [60].

### 3.7. Osteoarthritis

Related to osteoarthritis, chitosan–silica nanoparticles loaded with *Echinacea purpurea* ethanolic extract (encapsulation efficiency of 69.1% and loading capacity of 36.1%) were tested in obese male rats by oral gavage. These nanoparticles inhibited the expression of IL-1β, TNF-α, NF-κB P65, and IL-6 in the NF-κB pathway, and they reduced the expression of COX-2, Prostaglandin E2 (PGE2), iNOS, and NO. There was also an improvement in collagen II expression [61].

Cannabidiol-Loaded Solid Lipid Nanoparticles (entrapment efficiency of 95.16 ± 0.14% and drug loading of 2.36 ± 0.05%) were tested in human chondrocytes and macrophage cell lines, while indomethacin was used as a positive control. This nanosystem reduced reactive oxygen and nitrogen species and pro-inflammatory cytokines, such as TNF-α and IL-6, in the cell lines tested [62].

Lastly, on osteoarthritis, cannabidiol-loaded poly(lactic-co glycolic acid) copolymer (CBD-PLGA) nanoparticles (encapsulation efficiency of 88.4 ± 11.49% and drug loading of 22.1 ± 2.87%) were tested in LPS-induced rat chondrocytes. There were 4 groups: Control, LPS, LPS + Cannabidiol, and LPS + Cannabidiol PLGA nanoparticles. The nanosystem could effectively decrease the levels of inflammatory cytokines such as IL-1β, IL-6, TNF-α, and MMP-13 [63].

### 3.8. Rheumatoid Arthritis

In the context of rheumatoid arthritis, caffeic acid-9AA-conjugated nanomicelles (encapsulation efficiency of 79% and drug loading of 35%) were tested in Wistar rats, and there was an inhibition of the NF-κB pathway. There was also an activation of NR4A1 by 9AA, which led to the suppression of HIF-1α [64].

Also, luteolin was encapsulated with dexamethasone in hyalurosomes (luteolin encapsulation efficiency from 91.06 to 94.44%; dexamethasone encapsulation efficiency from 71.45 to 86.26%), and those were investigated in rats by topical application in the right knee. The rats were divided into 6 groups: saline (positive control), negative control, blank hyalurosomes, luteolin suspension, luteolin hyalurosomes, as well as luteolin and dexamethasone hyalurosomes. This nanosystem could reduce the serum levels of inflammatory markers such as TNF-α and IL-1β. In addition, there was a histological improvement of joint inflammation and swelling [65].

Quercetin–iron ultrasmall coordination nanoparticles were investigated in vitro in RAW 264.7 cells, and there was a removal of reactive oxygen species (ROS), reduction in cell apoptosis, inhibition of the NF-kB pathway, reduction in the pro-inflammatory M1 phenotype, and reduction in the pro-inflammatory cytokines release, such as TNF-α and IL-1β [66].

In addition, the same nanosystem was investigated in collagen-induced arthritis mice, through intra-articular injection into the foot joint. There were 3 groups: control, rheumatoid arthritis, and the Fe-Qur NCNs group, and it was demonstrated that it was able to reduce inflammatory cell infiltration, increase anti-inflammatory macrophage phenotypes—M2, and decrease M1. After treatment, many pro-inflammatory factors, such as TNF-α, IL-6, and IL-1β, were reduced, and IL-10, which has anti-inflammatory activity, was increased. It also showed excellent potential for ROS scavenging ability [66].

### 3.9. Skin Inflammation

Concerning skin inflammation, *Eupatorium japonicum* flavonoids-loaded gold nanoparticles (flavonoids loading of 5 mg/mL) were incorporated into HaCaT cells, compared to negative control (NC) cells, and there was an inhibition of pro-inflammatory cytokines, among which were IL-6 and IL-8, as well as downregulation of MAPK and NF-kB signaling pathways and intracellular reactive oxygen species [67].

*Cotyledon orbiculata*-loaded biogenic silver nanoparticles were investigated in mouse skin fibroblasts (KMST-6). Compared to the negative control (untreated cells), they could accelerate wound healing. In addition, there was downregulation of pro-inflammatory factors, such as CCL2, CXCL2, IL-6, MMP2, and SERPINE1, which proves that this system has anti-inflammatory properties [68].

In addition, Curcumin-loaded poly-(L-lactic acid) yarns (drug loading from 1 to 5%, *w*/*w*) were investigated in rats; there were 3 groups: Control, PLLA yarns, and Cur-PLLA yarns. The nanosystem provided good epithelialization, tissue regeneration, reduction in inflammation, and cellularity [69].

Gallic acid-loaded hyaluronic acid engineered chitosan nanoparticles (entrapment efficiency of 93.24 ± 0.132% and drug loading of 73.17 ± 0.23%) were tested and topically administered in mice. There were 3 groups: the first group was control, which received no treatment, the second group received formalin treatment, and the third group was treated with a gel loaded with the nanoparticles. That treatment resulted in targeting of CD44, which restricted keratinocyte hyperproliferation and reduced inflammation [70].

Furthermore, Kaempferol-loaded in situ electrospinning dressings were tested in diabetic rats. There were 5 groups: Control, gauze, and 3 portable electrospinning dressings (PEDs): PED0, PED2, and PED4 (the only one that had Kaempferol). The control group received no treatment; PED4 suppressed the expression of MMP9, promoted the polarization of macrophages from M1 to M2, reduced pro-inflammatory factors such as TNF-α, IL-1β, and IL-6, and upregulated IL-10, which is anti-inflammatory [71].

In addition, a cellulosic textile/clove (*Syzygium aromaticum*) nanocomposite was tested in rats. The animals were divided into two groups. The first group was a control group and did not receive any treatment, and the second group was the target group. The group that was treated with the nanosystem experienced an acceleration of wound healing and a reduction in the wound size [72].

Rutin-loaded ethosomes (encapsulation efficiency of 67.5 ± 5.2% and drug loading of 27 ± 1.7%) were tested and topically applied on the forearm of healthy human volunteers, compared with NaCl 0.9% (*w*/*v*) aqueous solution and a water–ethanol solution of rutin. With the nanosystem treatment, there was a reduction in the erythema index with physiological restoration of skin integrity [73].

*Hypericum perforatum* callus extract-loaded chitosan/alginate hydrogel was studied in vivo in healthy male BALB/c mice, through injection via the tail vein. There was accelerated re-epithelialization, neovascularization, collagen deposition, and reduction in inflammation [74].

### 3.10. Spinal Cord Injury

A system of arctigenin-loaded, cluster-like mesoporous silica/CAQK (entrapment efficiency of 31.21 ± 3.6% and drug loading of 1.56 ± 0.25%) was tested in C57BL/6J mice. The mice were injected with PBS, C-MSN, ARC-G, or MSN-FC@ARC-G. This system could induce a reduction in the expression of IL-17 and other pro-inflammatory cytokines: IL-1α, IL-6, G-CSF, MCP-1, and MIP-1β. There was also protection of neurons and promotion of recovery from spinal cord injury [75].

### 3.11. Ulcerative Colitis

Curcumin-loaded, laminarin-coated, folic acid-modified lactoferrin nanoparticles (curcumin loading of 95.08%) were studied in DSS-induced ulcerative colitis mice, orally. The mice were divided into six groups: normal group, model group, free curcumin group, curcumin nanoparticles group, folic acid curcumin nanoparticles group, and laminarin-coated folic acid curcumin nanoparticles group. The treatment with the nanosystem in the test inhibited the TLR4/NF-kB signaling pathway, which consequently led to a reduction in the expression of pro-inflammatory cytokines such as iNOS, IL-1β, IL-6, and TNF-α [76].

Isoliquiritigenin-loaded zein/caseinate nanoparticles (entrapment efficiency of 9.39 ± 0.26%) were studied in mice through gavage. The mice were divided into 5 groups: normal control, UC model group, free ISL-treated group, and ISL@NPs-treated group in two concentrations. The nanosystem demonstrated inhibition of pro-inflammatory factors, such as IL-6 and TNF-α, and there was a reduction in inflammatory cell infiltration in colon tissues, thereby promoting the repair of damaged intestinal mucosa [77].

A bruceine D self-nanoemulsifying drug delivery system (SNEDDS) (entrapment efficiency of 84.2 ± 1.5%) was studied in a TNBS-induced UC rat model, by oral gavage. The rats were randomly distributed into six groups: Normal control (intact), TNBS group (vehicle), positive group (AZA), BD-suspension group, and three doses of BD-SNEDDS groups. The treatment with this nanosystem inhibited pro-inflammatory cytokines such as TNF-α, IL-8, IL-1β, and IL-6 and restored the levels of IL-10 and TGF-β. In parallel, it suppressed NF-κB p65 and downregulated iNOS and COX-2 gene expression [78].

Lastly, gallic acid-loaded thiolated mucoadhesive anionic nanoliposomes (encapsulation efficiency of 78.91 ± 4.77% and drug loading of 13.23 ± 2.33%) were investigated in LPS-stimulated RAW 264.7 cells. The treatment was given in varying concentrations of free gallic acid and the gallic acid-loaded thiolated mucoadhesive anionic nanoliposomes. With this nanosystem, there was a decrease in the levels of pro-inflammatory cytokines such as IL-1β and TNF-α, as well as a reduction in nitrite production [79].

These nanoliposomes were also administered in a DSS-induced colitis mouse model. There were 4 groups: control, DSS-colitis, DSS + Th@APDL, and DSS + GATh@APDL. The nanosystem demonstrated an inhibition of the NF-kB pathway, a reduction in neutrophil and eosinophil levels, and a reduction in inflammatory lymphocytes, such as CD4+ helper T cells, CD8+ cytotoxic T cells, and B cells [79].

In the ambit of inflammatory bowel disease, nanoparticles with chlorogenic acid and mannose-modified chitosan were tested in RAW 264.7 cells. The cells were treated with either control medium, chlorogenic acid, chlorogenic acid nanoparticles, or chlorogenic acid nanoparticles @MCS. The concentrations of inflammatory cytokines, including TNF-α, IL-1β, and IL-6, were reduced, and the levels of IL-10 (anti-inflammatory) were increased. The nanosystem suppressed M1 polarization, alleviating inflammation [80].

The same nanosystem was tested in vivo in mice, orally. The mice were divided into 6 groups: control, DSS, chlorogenic acid, chlorogenic acid nanoparticles, or chlorogenic acid nanoparticles @MCS. The nanosystem could increase M1 macrophages and decrease M2 macrophages. It could also inhibit TLR4 NF-κB inflammatory pathway; therefore, they also decreased TNF-α, IL-1β, IL-6, and INOS and increased IL-10 [80].

Inulin-based nanoparticles with cannabidiol (encapsulation efficiency of 59.71 ± 0.71% and drug loading of 5.43 ± 0.06%) were tested in vitro in RAW 264.7 cells, and they could inhibit the levels of NO, iNOS, TNF-α, and IL-1β. In addition, they promoted the production of anti-inflammatory factors such as IL-4 and 1L10 [81].

This nanosystem was also tested in mice, orally; and they were divided into groups: control, DSS, 5-aminosalicylate + DSS, free CBD + DSS, and CBD NPs + DSS. The nanosystem, tested in the last group, ameliorated inflammation by modulating the TLR4 NF-κB signaling pathway. In addition, the activity of myeloperoxidase and the levels of inflammatory factors NO, iNOS, TNF α, and IL-1β were significantly decreased, while the levels of anti-inflammatory factors such as IL-4 and IL-10 were significantly increased [81].

Berberine-loaded, hyaluronic acid-modified chitosan–guanidine–CO_2_ nanoparticles (encapsulation efficiency of 61 ± 6.8% and drug loading of 2.9 ± 0.23%) were administered orally in mice. Compared to the treatments individually, the nanosystem caused ameliorated levels of CD98, TNF-α, IL-1β, and MPO activity. There was also a notable reduction in regional lymphocytic infiltration [82].

### 3.12. Neurodegenerative Conditions

In the context of neurodegenerative conditions, starch nanoparticles with cannabidiol (entrapment efficiency from 29.2 ± 10.6% to 39.4 ± 13.9%) were tested in vitro using the LPS-induced BV2 microglia cell line. In this study, they were able to reduce nitric oxide production and IL-6 levels [83].

In the context of Alzheimer’s Disease, lipid nanoparticles (LNPs) functionalized with mannose and penetratin (encapsulation efficiency ≥80%) were tested in LPS and in human Aβ_1–42_ oligomers. In this study, the nanoparticles reduced the protein and mRNA expression of pro-inflammatory cytokines TNF-α and IL-1β [84].

### 3.13. Other Inflammatory States

In the context of inflammation, American ginseng-loaded vesicle-like nanoparticles (AGVNs) were tested in vitro in RAW 264.7 macrophage cells, and they had an effect of decreasing inflammatory factors, including nitric oxide (NO), TNF-α, IL-6, and IL-10 [85].

This nanocarrier system was also studied in vivo with zebrafish. The fish were divided in 7 groups: control, model, ibuprofen, and AGVNs in 4 concentrations. The nanosystem showed anti-inflammatory activity, since it could reduce the number of inflammatory cells [85].

Moreover, *Cotyledon orbiculata* (commonly known as pig’s ear) was loaded into silver nanoparticles, and that system was studied in LPS-treated macrophages. The groups were as follows: Untreated, LPS control, and the nanosystem in study, and it could inhibit the secretion of pro-inflammatory cytokines such as TNF-α, IL-6, and IL-1β [86].

Luteolin–cerium ion nanocomplexes (luteolin loading of 82%) were investigated in vitro in RAW 264.7 cells, and they could scavenge the excess ROS, prevent cell apoptosis, downregulate inflammatory cytokine levels, regulate the response of inflammatory macrophages, and suppress the activation of the NF-kB pathway [87].

These nanocomplexes were also tested in vivo in Acute Kidney Injury (AKI) rats, and that resulted in an efficient improvement in kidney function, repair of damaged renal tissue, and reduction in oxidative stress, inflammatory response, and cellular apoptosis [87].

Furthermore, oridonin–Iridium (III) complexes were studied in P50 knockdown cells, and these complexes could detect the p50 subunit of NF-κB. In addition, they tracked NF-κB translocation induced by TNF-α, which can lead to important anti-inflammatory effects by modulation of the pathway [88].

Moreover, *Tinospora cordifolia*-loaded magnesium hydroxide nanoparticles were studied in vitro in a RAB1130-1KT ovine kit, while Ibuprofen was used as positive control. The nanoparticles induced inhibition of cyclooxygenase-1 (COX-1) and cyclooxygenase-2 (COX-2) [89].

In addition, oleic acid-loaded, lipid-based nanoparticles were incorporated into isolated human neutrophils, and they could inhibit superoxide and elastase production in activated neutrophils. This nanosystem was also tested in mice intravenously and it reduced myeloperoxidase (MPO) levels and cytokine production, such as TNF-α and IL-6. It also decreased pulmonary neutrophil recruitment and lung damage [90].

Lupeol-loaded and PEG-coated selenium nanoparticles with *Gymnema sylvestre* and *Cinammon cassia* were investigated orally in Sprague Dawley rats; the positive control was Diclofenac Potassium. With the treatment, there was a reduction in paw edema by reducing the release of pro-inflammatory factors [91].

Moreover, *Thymus vulgaris*-loaded selenium nanoparticles were investigated in a BSA protein denaturation assay to evaluate anti-inflammatory activity. Dimethyl sulfoxide was added to BSA solution and served as the control group. Through the nanosystem treatment, there was inhibition of the protein denaturation, which confirms anti-inflammatory activity [92].

In addition, *Panax ginseng*-loaded gold and silver nanoparticles were investigated in LPS-induced RAW 264.7 cells, and there was an inhibition of NO production [93].

Amphiphilic polymeric nanoparticles loaded with curcumin (encapsulation efficiency from 72 ± 4% to 79 ± 4%) were tested in LPS-induced RAW 264.7 cells, and there was a reduction in pro-inflammatory factors such as NO, IL-6, TNF-α, and MCP-1 [94].

Additionally, a crocin-loaded nanoformulation (entrapment efficiency of 54.66 ± 6.02% and drug loading of 1.89 ± 0.01%) was investigated using male Wistar rats, intraperitoneally. The rats were divided into control group, paraquat group, a crocin group, nano-crocin, crocin + paraquat, and nano-crocin + paraquat. With the nanosystem treatment, there was mitigation of paraquat-induced liver damage. There was also a reduction in the levels of TNF-α, IL-1β, NF-κB, and mRNA, as well as a decrease in LPO and ROS generation. In addition, there was attenuation of reduced thiol level and SOD activity [95].

Nanoliposomes loaded with lycopene (entrapment efficiency of 85.13 ± 3.78%) were investigated in rats with a high-fat diet (HFD). The control group was constituted by rats fed on the normal diet, the other group had a normal diet and was given Lip-Lyco, the NAFL group was fed on the HFD, and the other group was fed on the HFD diet and received Lip-lyco treatment. Orally, the nanosystem induced a reduction in the levels of pro-inflammatory cytokines such as TNF-α, IL-6, and IL-1β. There was also an inhibition of the production of COX-2 [96].

## 4. Conclusions

In summary, inflammation represents an extremely important target to investigate and develop therapies, since it plays a central role in a wide range of diseases. While conventional therapies are an option, the use of medicinal plants has gained increasing attention recently, offering a promising alternative to them. Nanotechnology represents a revolutionary approach in the scientific and therapeutic field, offering multiple significant strategies, and it must be explored to help overcome the difficulties and limitations that occur when using medicinal plants. Medicinal plants incorporated in nanodelivery systems have demonstrated efficacy in attenuating inflammation in various conditions through multiple mechanisms of action, and the present work provides scientific evidence supporting this idea.

## 5. Future Directions

In the future, it is expected that more nanosystems incorporating natural compounds will be developed, so it would be important that more systems were studied and tested.

Long-term toxicity represents an aspect that is not deeply studied and that must be studied more to understand possible consequences. In fact, perusing on NPs-induced potential toxicity on human health linking medicine and technology engineering would intensely contribute to promoting better nanotechnology application.

There is a problem associated with nanotechnology that consists of high costs in the production of drug delivery systems. It will be necessary to find a solution to reduce the costs, because the objective is that the nanosystems are produced industrially.

Therefore, it is relevant to study the scalability of the systems and evaluate them experimentally versus industrially, because these systems must be useful for the patients, and they have to be produced at an industrial level.

Artificial Intelligence (AI) plays a crucial role in bioactivity and biotechnology studies because it presents the fastest results without scientists having the need to try every possibility. It saves resources. Not only does it allow them to understand which nanosystems are worth producing, but it also allows them to understand against what diseases and conditions they are effective. Future research should prioritize the treatment of high-quality datasets encompassing the physicochemical properties of phytochemicals, nanocarrier descriptors, and bioactivity outcomes to support robust predictive modeling. Machine learning algorithms can then guide the nanocarrier types selection, encapsulation strategies, and formulation parameters, while optimization techniques such as Bayesian or genetic algorithms can efficiently balance multi-objective goals, including stability, encapsulation efficiency, and anti-inflammatory efficacy. AI-driven analysis of omics and high-throughput screening data may provide mechanistic insights into how nanoformulations modulate inflammatory pathways or immune cell behavior. Furthermore, predictive models for in vivo pharmacokinetics, biodistribution, and toxicity can reduce reliance on extensive animal testing, while integration with automated laboratory platforms allows rapid validation of high-performing formulations. Finally, AI can support regulatory readiness by monitoring batch consistency, predicting stability, and standardizing potency assays.

## Data Availability

No new data were created or analyzed in this study. Data sharing does not apply to this article.

## Appendix A

**Table A1:** Studies concerning anti-inflammatory properties of nanoplatforms with natural compounds.

Nanosystem	Model	Administration Route	Mechanism of Action	Ref.
***Nanocrystals***				
Crocin-loaded nanoformulation	Male Wistar rats	Intraperitoneal injection	Reduction in TNF-α, IL-1β, and NF-κB mRNA levels; Decrease in LPO and ROS generation; Attenuation of the reduced thiol level and SOD activity	[95]
***Inorganic nanoparticles***				
Capsaicin–Iron nanoparticles	LPS-induced RAW 264.7 cells	-	Decrease in the levels of TNF-α and iNOS; Increase in the levels of TGF-β	[48]
	C57 mice	Intraperitoneal injection	Decrease in the expression of pro-inflammatory factors such as TNF-α and IL-6	[48]
Quercetin–Iron ultrasmall coordination nanoparticles	RAW 264.7 cells	-	Removal of reactive oxygen species (ROS), reduction in cell apoptosis, and inhibition of the NF-kB pathway Reduction in the release of TNF-α and IL-1β	[66]
*Eupatorium japonicum* flavonoids-loaded gold nanoparticles	HaCaT cells	-	Inhibition of pro-inflammatory cytokines (like IL-6 and IL-8) and downregulation of MAPK and NF-κB signaling pathways	[67]
*Cotyledon orbiculata*-loaded biogenic silver nanoparticles	Mouse skin fibroblasts (KMST-6)	-	Downregulation of pro-inflammatory genes (CCL2, CXCL2, IL-6, MMP2, SERPINE1)	[68]
*Cotyledon orbiculata*-loaded silver nanoparticles	LPS-treated macrophages	-	Inhibition of the secretion of pro-inflammatory cytokines: TNF-α, IL-6 and IL-1β	[86]
Luteolin–Cerium ion nanocomplexes	RAW 264.7 cells	-	Scavenging of excess ROS; Prevention of cell apoptosis; Down-regulation of inflammatory cytokine levels; Regulation of the response of inflammatory macrophages; Suppression of the activation of the NF-κB pathway	[87]
	AKI rats	Intravenous injection	Improvement of kidney function; Repair of damaged renal tissue; Reduction in oxidative stress, inflammatory response, and cellular apoptosis	[87]
Oridonin–Iridium (III) complexes	P50 knockdown cells	-	Detection of the p50 subunit of NF-κB; Tracking of TNF-α-induced NF-κB translocation	[88]
*Tinospora cordifolia*-loaded Magnesium hydroxide nanoparticles	RAB1130-1KT ovine kit	-	Inhibition of COX-1 and COX-2	[89]
*Thymus vulgaris*-loaded Selenium nanoparticles	BSA bovine serum albumin	-	Inhibition of protein denaturation	[92]
*Panax ginseng*-loaded Gold and Silver nanoparticles	LPS stimulated RAW 264.7 cells	-	Inhibition of LPS-induced NO production	[93]
Naringenin-reduced graphene oxide nanosheets	Rats	Oral gavage	Decrease in serum levels of pro-inflammatory cytokines: IL-1β and IL-6; Amelioration of oxidative stress	[56]
Quercetin–Iron natural coordination nanoparticles	Collagen-induced arthritis (CIA) mice	Tail vein injection	Reduction in inflammatory cell infiltration, increase in anti-inflammatory macrophage phenotypes (M2) and reduction in pro-inflammatory M1; Inhibition of TNF-α, IL-6 and IL-1β; Increase in IL-10; ROS scavenging potential	[66]
Arctigenin-loaded cluster-like mesoporous silica/CAQK composite	C57BL/6J mice	Tail vein injection	Reduction in the expression of IL-17; Reduction in the expression of pro-inflammatory cytokines such as IL-1α, IL-6, G-CSF, MCP-1, and MIP-1β; Protection of neurons and promotion of recovery from spinal cord injury	[75]
Lupeol-loaded and PEG-coated Selenium nanoparticles with *Gymnema sylvestre*/*Cinnamon cassia* extracts	Sprague Dawley rats	Oral gavage	Reduction in edema by reducing the release of pro-inflammatory factors	[91]
***Polymeric nanoparticles***				
*Anacardium occidentale*-loaded chitosan/PVA/Copper oxide nanoparticles	COX-1 and COX-2 kits	-	Targeting and inhibition of the activity of COX-1 and COX-2	[15]
β-carotene-loaded hyaluronic acid-modified polycyclodextrins	HCEC (Human Corneal Epithelial Cells)	-	Decrease in reactive oxygen species and inflammatory factors such as TNF-α and IL-1β; Promotion of ocular surface mucins secretion	[59]
Cannabidiol-loaded poly(lactic-co glycolic acid) copolymer nanoparticles	LPS-induced rat chondrocytes	-	Decrease in the levels of IL-1β, IL-6, TNF-α and MMP13	[63]
Nanoparticles with chlorogenic acid and mannose modified chitosan	RAW 264.7 cells	-	Decrease in TNF-α, IL-1β, IL-6. Increase in IL-10; Modulation of macrophage M1/M2 polarization	[80]
Inulin-based nanoparticles with cannabidiol	RAW 264.7 cells	-	Reduction in the levels of NO, Inos, TNF-α and IL-1β; Increase in the production of IL-4 and IL-10	[81]
	Mice	Oral gavage	Modulation of TLR4 NF-Kβ pathway; Decrease in NO, INOS, TNF-α and IL-1β; Increase in IL-4 and IL-10 levels	[81]
Starch nanoparticles with cannabidiol	LPS-induced BV2 microglia cell line	-	Reduction in NO and IL-6 levels	[83]
Curcumin-loaded amphiphilic polymeric nanoparticles	Human articular chondrocytes RAW 264.7 cells	-	Reduction in pro-inflammatory factors: IL-8, MCP and MIP (chondrocytes); Reduction in nitric oxide, IL-6, TNF-α, and MCP-1 (RAW 264.7 cells)	[94]
*Aloe vera*-loaded chitosan nanoparticles	Rats	Intraperitoneal injection	Inhibition of pro-inflammatory cytokines such as IL-1β, TNF-α, IFN-γ, IL-6 and the transcription factor NF-kB; Increase in IL-10	[49]
β-carotene-loaded hyaluronic acid-modified polycyclodextrins	Rats	Ocular	Reduction in IL-1β and TNF-α levels; Restoration of ocular surface tissue; Reduction in oxidative stress levels	[59]
Licochalcone-A-loaded PLGA nanoparticles	Rabbits	Eye conjunctival sac instillation	Reduction in ocular inflammation score	[60]
*Echinacea* purpurea-loaded chitosan-silica nanoparticles	Obese male rats	Oral gavage	Inhibition of the expression of IL-1β, TNF-α, NF-κB p65 and IL-6 in the NF-κB pathway; Decrease the expression of COX-2, PGE2, iNOS and NO; Improvement of collagen II expression	[61]
Curcumin-loaded poly-(L-lactic acid) yarns	Male Wistar rats	Surgical suture	Reduction in inflammation and cellularity; Tissue regeneration	[69]
Gallic acid-loaded hyaluronic acid engineered chitosan nanoparticles	Mice	Topical	Targeting of CD44 receptors, restriction of keratinocyte hyperproliferation	[70]
Nanoparticles with chlorogenic acid and mannose modified chitosan	Mice	Oral	Polarization of macrophages towards M2; Inhibition of TLR4 NF-KB pathway; Decrease in TNF-α, IL-1β, IL-6; Increase in IL-10	[80]
Curcumin–cholesteryl–hyaluronic acid nanogel	Human pancreatic adenocarcinoma MiaPaCa-2 cells	-	Apoptosis and cytotoxicity in cancer cells by suppressing NF-κB, TNF-α, and COX-2	[53]
	Aggressive orthotropic murine mammary carcinoma 4T1 model	Intraperitoneal injection	Effective tumor growth inhibition	[53]
	Human pancreatic adenocarcinoma MiaPaCa-2 xenograft mice model	Intraperitoneal injection	Effective tumor growth inhibition	[53]
*Syzygium aromaticum*-loaded nanoemulsion	Rats	Topical	Acceleration on wound healing; Wound size reduction	[72]
*Hypericum perforatum* callus extract (HPCE)-loaded chitosan/alginate hydrogel	Healthy male BALB/c mice	Topical	Acceleration of re-epithelialization, neovascularization, and collagen deposition and reduction in inflammation	[74]
Curcumin-loaded, laminarin-coated, folic acid-modified lactoferrin nanoparticles	DSS-induced ulcerative colitis mice	Oral	Inhibition of the TLR4/NF-κB signaling pathway; Reduction in the expression of pro-inflammatory cytokines: iNOS, IL-1β, IL-6, and TNF-α	[76]
Isoliquiritigenin-loaded zein/caseinate nanoparticles	Mice	Oral	Inhibition of pro-inflammatory factors (IL-6 and TNF-α); Reduction in inflammatory cells infiltration in colon tissues; Repair of damaged intestinal mucosa	[77]
Bruceine D self-nanoemulsifying drug delivery system	TNBS-induced UC rat model	Intracolonic injection	Suppression of pro-inflammatory cytokines TNF-α, IL-8, IL-1β, and IL-6; Restoration of the levels of TNF-β and IL-10; Suppression of NF-κB, downregulation of iNOS and COX-2 gene expression	[78]
Gallic acid-loaded thiolated mucoadhesive anionic nanoliposomes	DSS-induced colitis mice model	Intra-rectal infusion	Inhibition of the NF-κB pathway Reduction in neutrophil and eosinophil levels; Reduction in lymphocytes	[79]
Berberine-loaded hyaluronic acid-modified chitosan-guanidine-CO_2_ nanoparticles	Mice	Oral gavage	Downregulation of CD98, TNF-α and IL-1β expression; Amelioration of MPO activity; Decrease in lymphocytic infiltration	[82]
Kaempferol in situ electrospinning dressings	Diabetic rats	Topical	Inhibition of MMP9; M2 macrophage polarization; Reduction in pro-inflammatory cytokine levels (TNF-α, IL-1β, and IL-6); Upregulation of anti-inflammatory cytokines (IL-10)	[71]
Naringin-loaded biomimetic nanoparticles	RAW 264.7 cells	-	Reduction in ROS production	[51]
	Mice	Intrathecal injection	Reduction in IL-6 and IL-1β; Relief of pulmonary edema; Polarization of macrophages towards M2	[51]
Ginsenoside C-K-loaded liver-targeted nanoparticles	HepG2 cells	-	Reduction in the expression of pro-inflammatory genes: cytokines like IL16, IL34 and chemokines like CXCL5, CCL26, and CCL16	[57]
	HFD induced NAFLD mice	Tail vein injection	Retardation of the development of steatosis and fibrosis; Protection of cardiac tissues from lipo-toxicity; Inhibition of the mTOR Pathway	[57]
***Lipidic nanoparticles***				
*Teucrium polium*-loaded solid lipid nanoparticles	Ntra-2 cancer cells	-	Decrease in the expression of IL-6 and IL-1β	[54]
Celastrol-loaded EMVs	RAW 264.7 cells	-	Modulation of macrophage M1/M2 polarization: suppression of M1 polarization and promotion of M2 polarization; Downregulation of the gene transcription of TNF-α, IL-1β, and iNOS; Upregulation of the gene transcription of Arg-1, Ym-1, and IL-10	[55]
	Mice	Tail vein injection	Modulation of macrophage M1/M2 polarization; Reduction in the levels of pro-inflammatory cytokines like TNF-α, IL-1β and iNOS; Prevention of neutrophil infiltration	[55]
Cannabidiol-loaded solid lipid nanoparticles	Human chondrocytes and macrophage cell lines	-	Reduction in reactive oxygen and nitrogen species; Reduction in pro-inflammatory cytokines: TNF-α and IL-6	[62]
Gallic acid-loaded thiolated mucoadhesive anionic nanoliposomes	LPS-stimulated RAW 264.7 cells	-	Decrease in the levels of pro-inflammatory cytokines (IL-1β and TNF-α) and nitrite levels	[79]
Lipid nanoparticles with mannose and penetratin	LPS and human Aβ_1–42_ oligomers	-	Reduction in TNF-α and IL-1β	[84]
American ginseng-loaded vesicle-like nanoparticles (AGVNs)	RAW 264.7 cells	-	Reduction in inflammatory factors such as NO, TNF-α, IL-6, and IL-10	[85]
Oleic acid-loaded lipid-based nanoparticles	Isolated human neutrophils	-	Inhibition of superoxide and elastase production in activated neutrophils	[90]
Auraptene-loaded nanostructured lipid carrier	Rats	Oral	Reduction in the expression of inflammatory markers such as NF-κB, IL-1β, IL-6, and TGF-β; Enhancement of antioxidant activity	[52]
Bryostatin-1-loaded engineered extracellular vesicles	EAE mice	Intravenous injection	Decrease in infiltration of pro-inflammatory cells Protection of blood–brain barrier Alteration of the microglia pro-inflammatory phenotype	[58]
Caffeic acid-9AA-conjugated nanomicelles	Wistar rats	Intradermal injection	Inhibition of NF-κB signaling pathway; Activation of NR4A1, which led to the suppression of HIF-1α	[64]
Dexamethasone and luteolin co-encapsulated hyalurosomes	Rats	Topical	Reduction in serum levels of TNF-α and IL-1β	[65]
Rutin-loaded ethosomes	Human healthy volunteers	Topical	Reduction in the erythema index with physiological restoration of skin integrity	[73]
American ginseng vesicle-like nanoparticles (AGVLN)	Zebrafish	Topical	Decrease in the number of inflammatory cells	[85]
Oleic acid-loaded lipid-based nanoparticles	Mice	Intravenous injection	Reduction in myeloperoxidase (MPO) levels and cytokine production (TNF-α and IL-6); Decreased pulmonary neutrophil recruitment and lung damage	[90]
Lycopene-loaded nanoliposomes	Rats with high-fat diet	Oral	Reduction in the levels of pro-inflammatory cytokines such as TNF-α, IL-6, and IL-1β; Inhibition of the production of COX-2	[96]
Curcumin platelet-derived nanoparticles	Mice	Inhalation	Decrease in lung vascular permeability and reduction in pro-inflammatory cytokine levels like TNFα, IL-6, ICAM1 and iNOS; Polarization towards the M2 subtype	[50]
